# Berberine alleviates pyroptosis of retinal ganglion cells in diabetic retinopathy by regulating AKT1, JUN, and STAT3

**DOI:** 10.1002/ibra.70018

**Published:** 2026-03-26

**Authors:** Na Li, Ji‐Lin Chen, Yi‐Jian Sun, Jia‐Fan Sun, Fatin Athirah Pauzi, Song‐Lin Zhu, Amy Yi Hsan Saik, Alan Han‐Kiat Ong

**Affiliations:** ^1^ M. Kandiah Faculty of Medicine and Health Sciences Universiti Tunku Abdul Rahman Kajang Malaysia; ^2^ Institute of Neuroscience Kunming Medical University Kunming China

**Keywords:** berberine, cell pyroptosis, diabetic retinopathy, network pharmacology

## Abstract

Berberine (BBR) exerts an effective protection for diabetic retinopathy (DR), but the underlying key molecular mechanism remains unknown; this study investigated the protective mechanism of BBR on DR by alleviating cell pyroptosis. A rat DR model was established and treated with BBR, and histological analyses, including hematoxylin and eosin staining, Nissl staining, and immunofluorescence, were executed to evaluate tissue changes. Core target genes were identified using the GeneCards database, Venn diagram analysis, Gene Ontology (GO), Kyoto Encyclopedia of Genes and Genomes (KEGG) pathway enrichment, protein‐protein interaction networks, and molecular docking. Validation of key genes was performed via reverse transcription‐quantitative polymerase chain reaction (RT‐qPCR), Western blot, and RNA interference. BBR improved retinal morphology, reduced edema, and restored the arrangement of retinal ganglion cells in DR rats. BBR significantly reduced the levels of pyroptosis markers such as IL‐1β and IL‐18, which were elevated in DR. Network pharmacology identified 10 hub genes, with six genes (*JUN*, *STAT3*, *AKT1*, *TP53*, *IL‐1B*, *EGFR*) further analyzed. BBR reversed DR‐induced upregulation of *JUN*, *STAT3*, and *AKT1* at both the mRNA and protein levels, as confirmed by RT‐qPCR and Western blot. Silencing these genes enhanced cell viability and amplified BBR's protective effects. Altogether, BBR alleviates retinal inflammation and pyroptosis in diabetic retinal ganglion cells by targeting *JUN*, *STAT3*, and *AKT1*, providing insights into its therapeutic potential for DR.

## Graphical abstract

Berberine alleviates diabetic retinopathy by reducing pyroptosis through AKT1/JUN/STAT3 regulation, as validated in rat models via network pharmacology, molecular docking, and experimental assays.

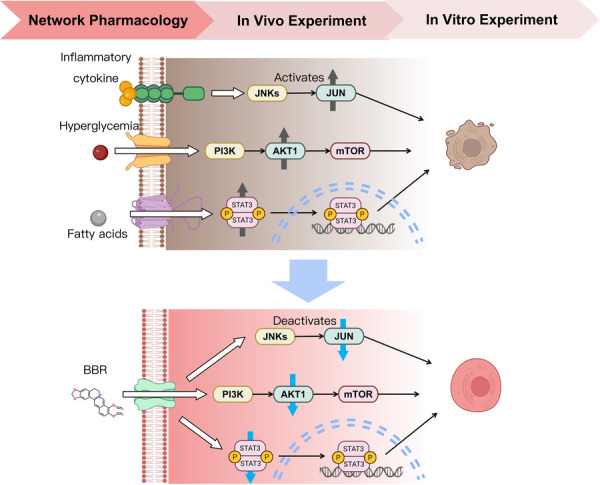

## 1 INTRODUCTION

Diabetic retinopathy (DR) is a microvascular complication deriving from diabetes and progressing to be the leading cause of blindness.^1^, ^2^ Microvascular complications in DR may induce cell pyroptosis, a chronic progressive disorder of neurovascular interdependent units (NVUs) that is associated with the pathogenesis of DR. NVUs consist of neurons, glial cells, and vascular cells,^3^ forming a vital structural network through their interdependencies, which becomes disrupted in diabetic eye conditions. The visually threatening stages of DR are characterized by clinically significant diabetic macular edema and proliferative DR, including traction retinal detachment and vitreous hemorrhage. According to the International Diabetes Federation Diabetes Atlas, the global prevalence of DR among individuals with diabetes is approximately 15.3–42.4%,^4^ but it is expected to increase along with the prevalence of diabetes, which is estimated to reach 10.2% (578 million) by 2030 and 10.9% (700 million) worldwide by 2045. It is reported that a lower age of type 2 diabetes onset was associated with a higher risk of retinopathy.^5^ The cumulative rate of any DR reached 47.26% among individuals with type 1 diabetes mellitus, reflecting a yearly occurrence of 15.16%; by comparison, patients with type 2 diabetes mellitus showed a total rate of 26.49%, along with an annual occurrence of 8.13%.^6^ Annual burden of DR reinforces the critical importance of timely screening, glycemic control, and targeted interventions to curb its escalating global impact.

Cell pyroptosis is a programmed pro‐inflammatory cell death mode characterized by an early breach of the plasma membrane integrity.^7^ The classical cell pyroptosis signaling pathway relies on NOD‐like receptor protein 3 (NLRP3) inflammasome and other inflammatory bodies to activate Caspase‐1, while the non‐classical cell pyroptosis pathway depends on Caspase‐11 (in mice) or Caspase‐4/5 (in humans), with both pathways ultimately leading to cell pyroptosis through their common effector substrate Gasdermin D (GSDMD). Though it has been known that pyroptosis can be triggered by pathogenic microbial infection or other danger signals, the exact underlying molecular mechanism remains unknown. Under high glucose conditions, endothelial cells and microglia can serve as sources of inflammatory factors such as interleukin (IL)‐1beta (IL‐1β), which may activate nuclear factor kappa‐light‐chain‐enhancer of activated B cells (NF‐κB) to induce pericellular pyroptosis.^8^ The molecular pathway of pyroptosis primarily involves the activation of caspase‐1, which subsequently processes IL‐1β and IL‐18 into their mature forms, leading to inflammatory response recruitment. Recent studies have shown that targeting this pathway, particularly through the inhibition of NLRP3 inflammasome activation, could be therapeutically beneficial, in the improvement of DR,^9^ or traditional Chinese medicine, may provide additional effective treatment options.^10^

Berberine (BBR) is a yellow‐colored bioactive compound, occurring naturally as a secondary metabolite in some plants, including species of Berberis, from which its name is derived. In addition, BBR, as a Chinese medicine, commonly used as an anti‐infective and anti‐inflammatory agent for gastrointestinal diseases such as diarrhea.^11^ Perhaps more importantly, the therapeutic effects of BBR in regulating glucose and lipid metabolism have been proven in numerous animal studies and clinical trials,^12^, ^13^ with its perspective potential in treating DR been constantly highlighted. Previous study in animal models had shown that BBR can improve β‐cell function in diabetes mellitus, lowering lipid level and regulating the expression of transcription factors including GSDMD(R), IL‐18(R), IL‐1β(R) and caspase‐1.^14^ Earlier studies have established that the BBR treatment protocol typically involves oral gavage administration at a dose of 100 mg/kg body weight daily for a duration of 1 month.^15^ In addition, BBR could protect against oxidative stress‐induced damage in animal models^1^ and inhibit cell death by upregulating oxidative stress in the cell model.^16^

Network pharmacology involves the network analysis of genes, drug targets, and diseases, with emphasis on the genes that drugs interact with in diseases and the regulation of signal pathways.^17^ In this study, network pharmacology analysis was employed to identify the core target genes of BBR in DR treatment, followed by experimental validation of these predictions, and crucial functional genes were screened.

## 2 METHODS

### 2.1 Animal and grouping

Adult male Sprague‐Dawley (SD) rats, aged 6–8 weeks and weighing approximately 220 g, were obtained from Kunming Medical Animal Center. They were divided into three groups: Normal, DR, and BBR groups, 15 rats in each group. The DR group received an intraperitoneal injection of streptozotocin (STZ) (60 mg/kg),^18^ After 72 h of STZ injection, tail venous blood glucose levels were measured, and rats with blood glucose levels ≥16.7 mmol/L were considered diabetic models and included in the DR and BBR groups. After the successful establishment of the DR model, the BBR group was given BBR via oral gavage administration at a dose of 100 mg/kg daily for 1 month.^15^

The rats were housed under controlled conditions (20°C–25°C, 45%–65% humidity) and fed nutrient‐rich diets daily. Body weight and blood glucose levels were recorded weekly during the 1‐month treatment period. At the end of the experiment, rats were euthanized by cervical dislocation.

After fixation with 4% paraformaldehyde (PFA/PBS) at 4°C for 24 h, the eyeball tissues were immersed in 10% (about 7 h), 20% (about 4 h), and 30% (about 12 h) sucrose solutions, respectively, until the tissue specimens completely sank in each solution. Then the tissue was embedded with optimal cutting temperature compound at −80°C and frozen for 30 min and performed a coronal section at 10 µm thickness.

All procedures were performed in accordance with the guidelines and approval of the Ethics Committee of the Kunming Medical University (Approval number is KMMU20242021). The application for ethical approval for research projects using animals (doctoral projects) has been approved by the UTAR Scientific and Ethical Review Committee (SERC) with the approval number Re: U/SERC/58‐29/2024.

### 2.2 Morphological staining

#### 2.2.1 H&E and Nissl staining

All sections from the DR group, Normal group, and BBR group were dried in an oven at 37°C for 10 min. For Hematoxylin‐eosin (H&E) staining, sections were washed three times with Phosphate Buffered Saline with Tween‐20 (PBST) for 1 min each. The tissue was first treated with hematoxylin stain for 5 min, then rinsed with tap water, followed by staining with eosin for 3 min., and soaked in water for 5 min. Sections were then dehydrated with graded ethanol (70%, 80%, 90%, 100%) for 1 min each, cleared in xylene for 3 min, and sealed with neutral gum for cell morphology observation.

For Nissl staining, sections were similarly dried at 37°C for 10 min and washed with PBST three times for 1 min each. Nissl staining solution (Servicebio, Wuhan, China) was applied at 37°C for 3–5 min, followed by washing with distilled water. Sections were dehydrated in graded ethanol (70%, 80%, 90%, 100%) for 1 min each, cleared in xylene for 3 min, sealed with neutral gum, and observed under a microscope.

#### 2.2.2 The immunofluorescence detection of IL‐1β, IL‐18, GSDMD, Caspase‐1 and NLRP3

PBST was used to wash the sections for three times for 1 min each. Next, the sections were incubated at room temperature for 3 h in 3% goat serum prepared in 0.3% Triton‐PBS solution. Primary antibodies including IL‐1β (#PB0055, 1:200, Boster Biological Technology), IL‐18 (#PB0058, 1:200, Boster Biological Technology), GSDMD (#A17308, 1:100, Abclonal Technology), Caspase‐1 (#BA2220, 1:200, Boster Biological Technology) and NLRP3 (#BA3677,1:200, Boster Biological Technology) and were incubated with tissue sections at 4°C overnight. After PBST washing, sections were incubated with Dylight488‐conjugated goat anti‐rabbit IgG secondary antibody (1:200, Hunan Aifen Biotechnology) at 4°C for 1 h, followed by PBS washing. Sections were then counterstained with DAPI (1:300), sealed, and observed under the fluorescence microscope. Immunofluorescence images were analyzed using ImageJ software. Five random fields from three independent sections per group were captured. The mean fluorescence intensity was measured and normalized to controls.

### 2.3 Network pharmacology analysis and molecular docking

Genes related to “diabetic retinopathy,” “berberine,” and “pyroptosis” were retrieved from the GeneCards database (https://www.genecards.org/), respectively. The overlapping genes among the three gene sets were analyzed using Venny2.1.0 (https://bioinfogp.cnb.csic.es/tools/venny/index.html), and a Venn diagram was constructed to visualize the intersecting genes.

The intersecting genes were further analyzed using the DAVID bioinformatics platform (https://davidbioinformatics.nih.gov/) for Gene Ontology (GO) and Kyoto Encyclopedia of Genes and Genomes (KEGG) pathway enrichment. The results were visualized in GO and KEGG enrichment analysis charts generated using the bioinformatics platform (http://www.bioinformatics.com.cn/).

A protein‐protein interaction (PPI) network was constructed for the intersecting genes using the STRING database (https://cn.string-db.org/), and associated proteins were analyzed. The PPI data were imported into Cytoscape 3.8.2 software to identify the top 10 hub genes.

Finally, the structures of proteins encoded by the identified genes were retrieved from the Protein Data Bank (PDB) (https://www.rcsb.org), and the structures of the drugs were obtained from the PubChem database (https://pubchem.ncbi.nlm.nih.gov). Molecular docking was performed using AutoDock, while Pymol and Open Babel were used to refine the protein and ligand structures. Hydrogen bonds were calculated, and the overall structures and ligand molecules were exported.^19^

### 2.4 RT‐qPCR‐based gene expression and validation of upstream signaling molecules using Western blot

A total of 3 samples from each group were prepared, and the tissues in each sample were subjected to total RNA extraction. Reverse transcription quantitative polymerase chain reaction (RT‐qPCR) was used to detect the gene expression of epidermal growth factor receptor (*EGFR*), *IL1B*, Jun proto‐oncogene, AP‐1 transcription factor subunit (*JUN*), signal transducer and activator of transcription 3 (*STAT3*), AKT serine/threonine kinase 1 (*AKT1*), and tumor protein p53 (*TP53*). Total RNA was extracted using the RNA extraction kit (Burlington Fermentation, Canada). Reverse transcription was performed using the reverse transcription kit (DBI Bioscience, Cat. No. 2022) according to the manufacturer's protocol. PCR amplification was conducted with the PCR amplification kit (DBI Bioscience, Cat. No. 2043) under the conditions: 94°C for 15 s, 55°C for 30 s, and 60°C for 60 s, for 45 cycles. GAPDH was used as the internal control. The mRNA expression levels of *EGFR*, *IL1B*, *JUN*, *STAT3*, *AKT1*, and *TP53* were analyzed using the 2^−ΔΔCt^ method. All primer for each gene were listed in Table 1.

**Table 1:** Primer sequences for the RT‐qPCR.

Name	Base sequence
*TP53:F*	CCTTACCATCATCACGCTGGAAGAC
*TP53:R*	AGGACAGGCACAAACACGAACC
*IL‐1beta:F*	GGGATGATGACGACCTGCTA
*IL‐1beta:R*	GGGATGATGACGACCTGCTA
*AKT1F:*	CATGAACGAGTTTGAGTACCT
*AKT1R:*	CTCCTTCTTGAGGATCTTCAT
*JUN F:*	CAACATGCTCAGGGAACAGGT
*JUN R:*	GTTTGCAACTGCTGCGTTAG
*STAT3F:*	CATGGAAATCAGACAGTACCT
*STAT3R:*	GAAAAGCGGCTGTACTGGTC
*EGFR:F*	AGAGAGTGACTGTCTGGTCT
*EGFR:R*	GTGGCACCAAAGCTGTACTT
*GAPDHF:*	AAGTTCAACGGCACAGTCAAG
*GAPDHR:*	CATACTCAGCACCAGCATCAC

Western blot analysis was conducted to examine the protein expression levels of AKT1, JUN, and STAT3. Cells or tissues were lysed in ice‐cold radioimmunoprecipitation assay (RIPA) buffer supplemented with protease and phosphatase inhibitors, followed by centrifugation at 12,000 rpm for 15 min at 4°C. Protein concentrations were measured using a bicinchoninic acid (BCA) protein assay kit. Equal amounts of protein were separated by sodium dodecyl sulfate‐polyacrylamide gel electrophoresis and transferred onto polyvinylidene fluoride (PVDF) membranes using a wet transfer system. Membranes were blocked with 5% non‐fat milk for 1 h at room temperature, incubated overnight at 4°C with primary antibodies specific to AKT1 (#ab192623, 1:1000, Abcam, Rabbit), JUN (#GB11515, 1:1000, Servicebio, Rabbit) and STAT3 (#69713, 1:1000, Abclonal, Rabbit) and β‐actin (#EM21002, 1:5000, Huaan Biotech, Rabbit). After the membrane was then washed with PBST, it was incubated with horseradish peroxidase (HRP)‐conjugated secondary antibodies IgG (#A21020, 1:10000, Abbkine, Rabbit) for 1 h. Enhanced chemiluminescence (ECL) reagents were used for signal detection and protein band intensities were analyzed using densitometric software, normalized to β‐actin as an internal control.

**Table 2:** Cell experimental grouping.

Group	Experimental group name
1	Blank (Normal group)
2	HG (high glucose) model group
3	Berberine treatment group
4	Berberine + NC‐siRNA group
5	Berberine + AKT1‐siRNA group
6	Berberine + JUN‐siRNA group
7	Berberine + STAT3‐siRNA group

### 2.5 Cell culture and cellular hyperglycemic model preparation

Retinal ganglion cells (RGCs) used in the experiment are sourced from BLUEFBIO™ Company (Code: BFN608006986). RGCs were recovered from liquid nitrogen storage and cultured in DMEM/F12 medium (Gibco, USA; 11320033) supplemented with 10% FBS and 1% penicillin‐streptomycin (Beijing Solepol; P1400). After rapid thawing at 37°C, cells were centrifuged (1000 rpm, 5 min, RT), resuspended in complete medium, and seeded in T25 flasks. Cells were maintained at 37°C in a humidified atmosphere containing 5% CO_2_ (thermos, 311), with medium changes every 3 days. Cell morphology was monitored using an inverted microscope (Nikon, CSIM100). Cell grouping is shown in Table 2.

RGCs at 80% confluence were seeded in 96‐well plates at 1 × 10^5^ cells/mL (100 μL/well) with seven groups. Cells were cultured in DMEM/F12 medium (Gibco; 11320033) supplemented with 10% fetal bovine serum (FBS) and 1% penicillin‐streptomycin. When the cells reached 60% confluence, they were exposed to high glucose (30 mmol/L) for 7 h at 37°C in 5% CO_2_ atmosphere.

Cells in the high glucose (HG) group were exposed to high‐glucose Dulbecco's Modified Eagle Medium/Nutrient Mixture F‐12 (30 mmol/L) at 37°C, 5% CO₂ for 7 h, followed by normal medium for 48 h. For the BBR intervention, high‐glucose‐induced cells were exposed to BBR (20 μM) treatment for 48 h. To investigate the underlying mechanisms, cells were co‐treated with BBR and specific siRNAs targeting *AKT1*, *JUN*, or *STAT3* after high glucose exposure. Small interfering RNA (siRNA) transfection was carried out using Lipofectamine RNAiMAX (Thermo Fisher Scientific). The final concentration of siRNA was 50 nM. After 24 h of transfection, the culture medium was replaced, and then the cells were treated with 20 μM BBR for 48 h. Cells cultured in normal medium served as the control, and the negative control siRNA was used to validate the specificity of RNA interference (RNAi). RGCs were seeded in 96‐well plates at 1 × 10^5^ cells/mL (100 μL/well) and cultured until 60% confluence. Following treatments, cell morphology and number were evaluated using an inverted microscope, with three random fields captured per well. Cell counts and morphological changes were compared between experimental and control groups.

### 2.6 CCK‐8 cell viability assay

RGCs were grown to 80% confluence and seeded into 96‐well plates at 1 × 10^5^ cells/mL (100 μL/well) with 7 experimental groups (*n* = 5 wells per group). Cells were maintained in DMEM/F12 complete medium at 37°C with 5% CO_2_ until reaching 60% confluence. Following experimental treatments, CCK‐8 reagent was added to each well and incubated for 2 h. The optical density was measured at 490 nm using a microplate reader (Varioskan LUX, Thermo) for statistical analysis.

### 2.7 TUNEL staining

When RGCs reached 80% confluence, cells were seeded into 96‐well plates (1 × 10^5^ cells/mL, 100 μL/well) with seven groups with five wells per group and cultured in DMEM/F12 complete medium (37°C, 5% CO_2_). At 60% confluence, cells were washed twice with PBS, fixed with 4% paraformaldehyde (30 min), and permeabilized with 0.3% Triton X‐100 (5 min, room temperature). After washing, cells were incubated with TUNEL detection solution (50 μL/well, 37°C, 60 min), washed three times with PBS/HBSS, mounted with anti‐fade medium, and observed under fluorescence microscopy.

### 2.8 Statistical analysis

All data were analyzed using SPSS 17.0 statistical software and presented by mean ± standard deviation (SD). The one‐way analysis of variance was used to compare the differences among the various groups. *p* < 0.05 was regarded as statistically significant.

## 3 RESULTS

### 3.1 Morphology findings

**Figure 1:**
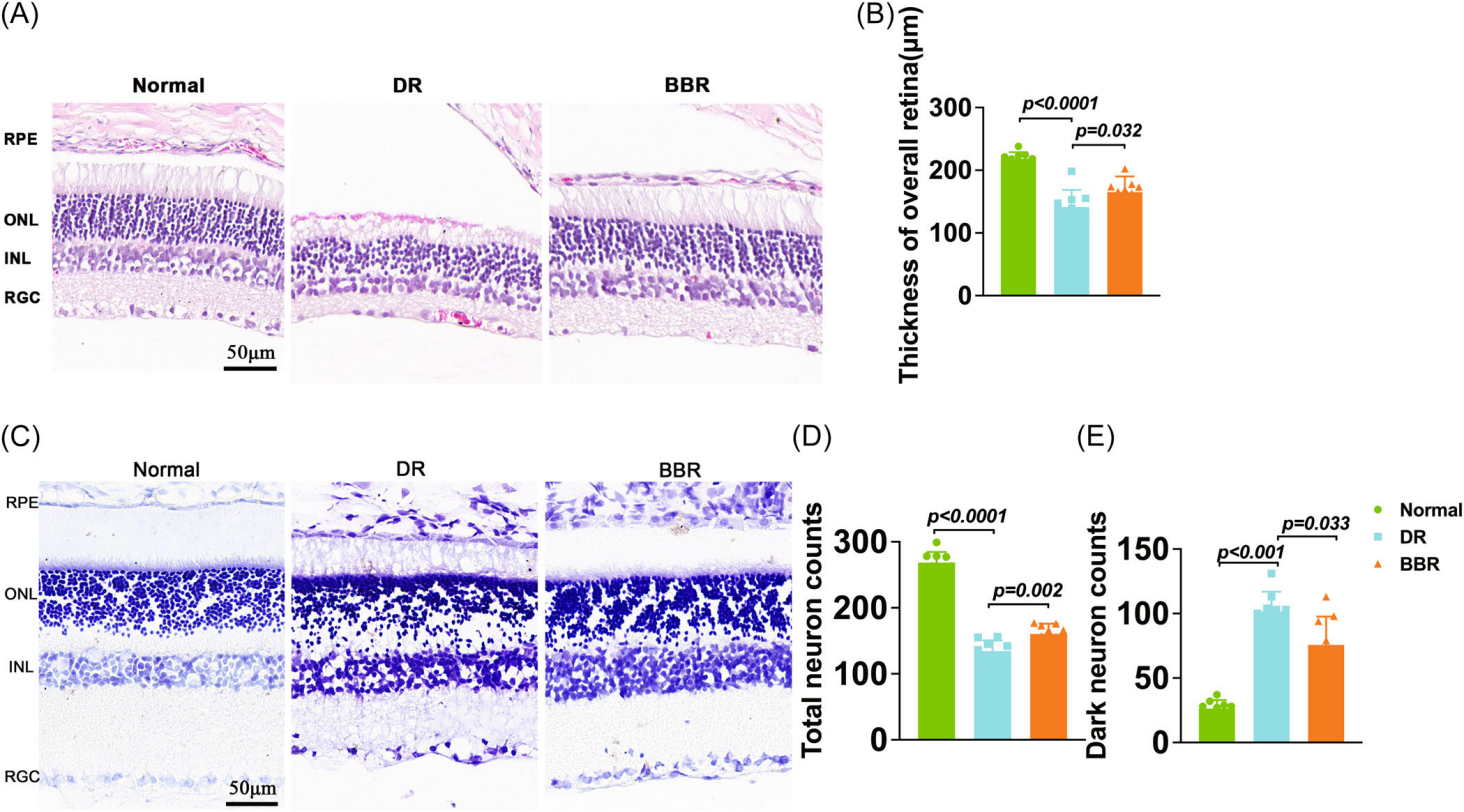
Hematoxylin and eosin (H&E) and Nissl staining in retina from each group (400'). (A, B) H&E staining and quantitative analysis. (C–E) Nissl staining and quantitative analysis. *n* = Nine sections from three rats per group. BBR, berberine; DR, diabetic retinopathy; INL, inner nuclear layer; ONL, outer nuclear layer; RPE, retinal pigment epithelium; RGC, retinal ganglion cell. Scale bar = 50 μm.

H&E staining reported that all retinal layers of rats in the Normal group were intact and neatly arranged, with the normal cell morphology within the inner boundary membrane, and the RGCs were arranged in a single layer within the ganglion cell layer (Figure 1A). Comparatively, in the DR group, the cells were disordered in each layer, and the boundaries were not clear. Meanwhile, dilated blood vessels were seen in the ganglion cell layer with vascular‐like structure, and the number of cells in the inner and outer nuclear layers was reduced and the arrangement was sparse. Compared with the DR group, the cells in the BBR group were arranged neatly and the ganglion cell layer edema was markedly reduced. Moreover, the retinal thickness was markedly decreased in DR group (*p* < 0.0001), while there was a significant increase in the BBR group (*p* = 0.032) (Figure 1B).

Nissl staining exhibited a similar trend to that of the H&E observation, which showed the retinal structure of the Normal group was clear and the cells of each layer were arranged neatly and closely (Figure 1C). However, in the DR group, the number of ganglion cells decreased gradually (*p* < 0.0001), and the distribution was disordered. Whereas, in the BBR group, the cell edema of each layer decreased significantly; the arrangement tended to be regular, and the distribution was orderly. Moreover, the number of RGCs in retinas increased markedly in the BBR group compared to that of the DR group (*p* = 0.002) (Figure 1D). Morphological analysis of dark neurons in retinal sections revealed that the number of dark neurons in the DR group was significantly increased (*p* < 0.001) while that of BBR group was reduced compared to the DR group (*p* = 0.033, Figure 1E), suggesting that BBR treatment partially alleviates the presence of dark neurons in the diabetic retina. This indicates that the therapeutic effects of BBR may involve the reduction of dark neuron counts and the morphological recovery of retinal neurons.

### 3.2 Immunofluorescence findings

**Figure 2:**
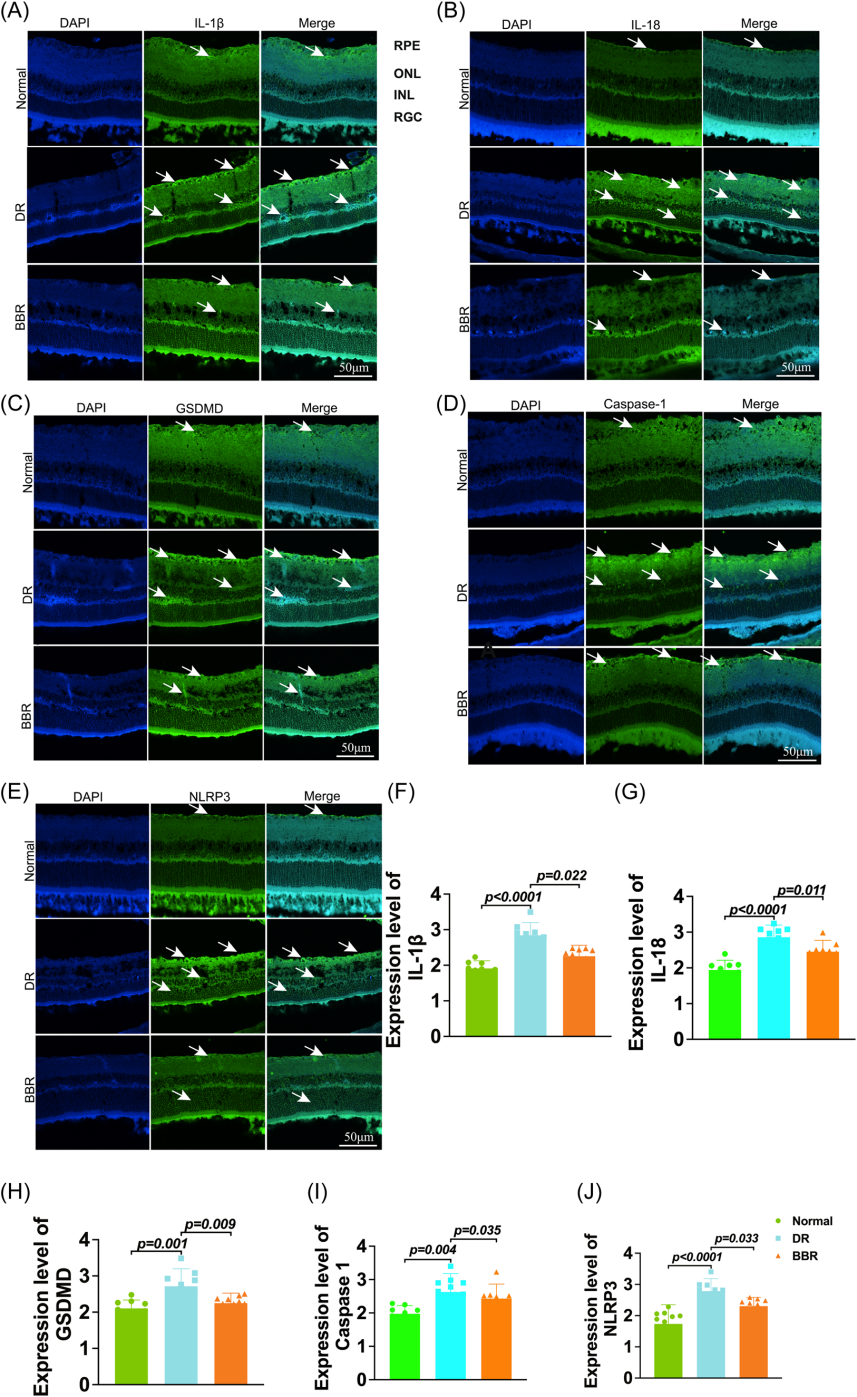
Immunofluorescence staining of inflammatory and pyroptosis markers. (A–E) Representative retinal immunofluorescence images showing IL‐1β, IL‐18, GSDMD, Caspase‐1, and NLRP3 expression in Normal, DR, and BBR groups. (F–J) Quantitative analysis of marker expression levels. *n* = 9 sections from three rats per group. BBR, berberine; DR, diabetic retinopathy.

Immunofluorescent staining revealed that the fluorescence intensity of IL‐1β, IL‐18, GSDMD, Caspase‐1, and NLRP3 in the retina was significantly increased in the DR group compared to the Normal group, with statistical significance (IL‐1β: *p* < 0.0001, IL‐18: *p* < 0.0001, GSDMD: *p* = 0.001, Caspase‐1: *p* = 0.004, NLRP3: *p* < 0.0001). BBR treatment effectively reduced the levels of these pyroptosis‐related markers in the retina compared to the DR group (IL‐1β: *p* = 0.022, IL‐18: *p* = 0.011, GSDMD: *p* = 0.009, Caspase‐1: *p* = 0.035, NLRP3: *p* = 0.033). Specifically, the fluorescence intensity of IL‐1β, IL‐18, GSDMD, Caspase‐1, and NLRP3 showed marked elevation in the DR group compared to the Normal group, while BBR treatment attenuated their levels significantly, demonstrating its therapeutic effect (Figure 2).

### 3.3 Network pharmacology analysis and molecular docking

#### 3.3.1 Network pharmacology analysis

**Figure 3:**
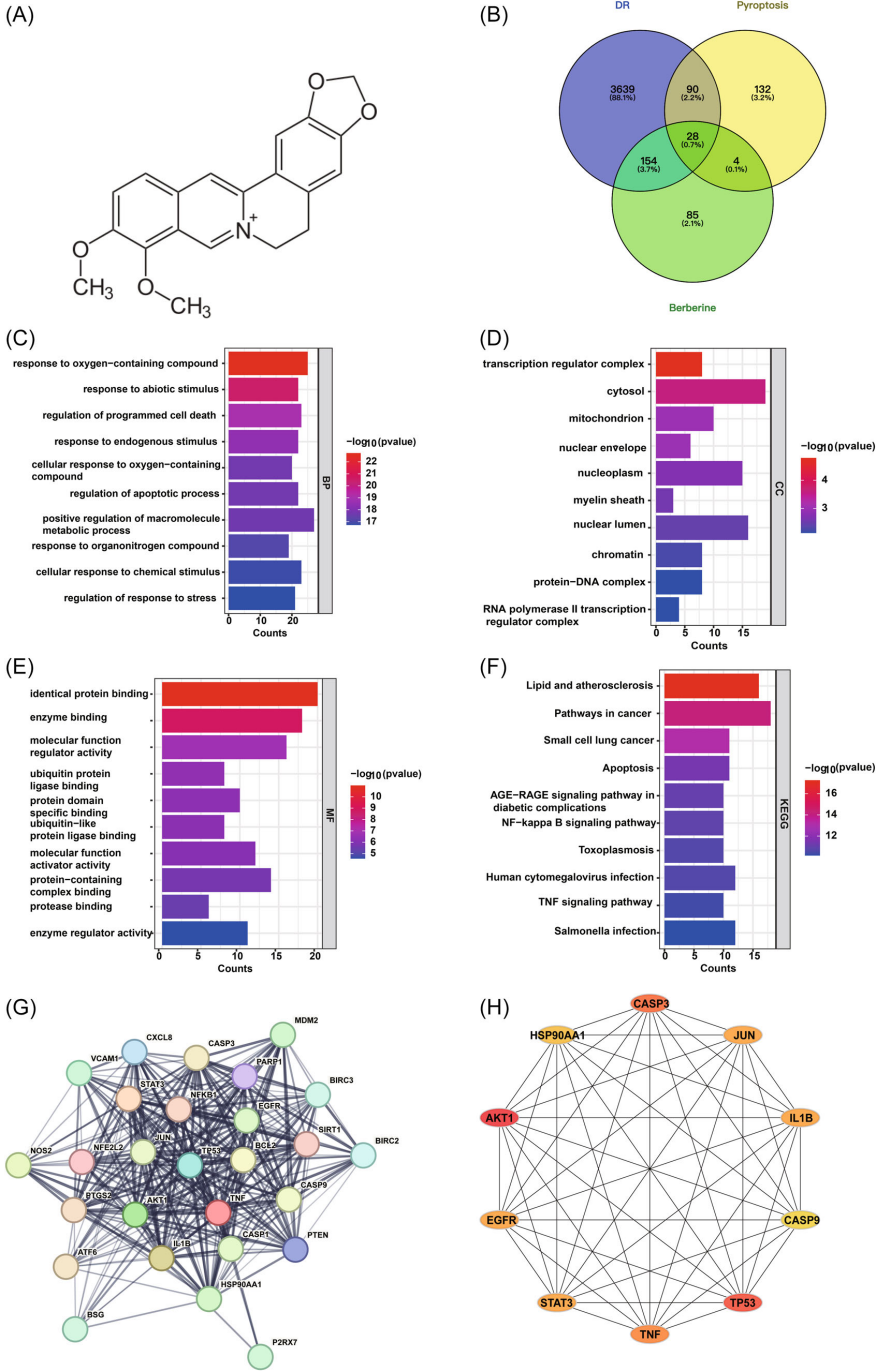
Network pharmacology analysis. (A) Chemical structure of BBR. (B) Venn diagram of BBR‐, DR‐, and pyroptosis‐related genes. (C) Top 10 BP terms. (D) Top 10 CC terms. (E) Top 10 MF terms. (F) KEGG pathway analysis reveals the top 10 pathways. (G) PPI network showing interactions among identified genes. (H) Hub genes with higher connectivity. BBR, berberine; BP, biological process; CC, cellular component; DR, diabetic retinopathy; MF, molecular function.

Chemical structure of BBR showing its protoberberine backbone, quaternary ammonium nitrogen, two methoxy substituents, and one methylenedioxy group (Figure 3A). The DR, BBR, and pyroptosis genes were then retrieved from GeneCards. In total, 3,911 DR‐related genes, 271 BBR‐associated genes, and 254 pyroptosis‐related genes were identified (Supplementary Tables 1–3). Subsequently, 28 intersection genes were obtained, namely, *TNF, MIR21, STAT3, IL1B, NOS2, AKT1, VCAM1, TP53, CXCL8, PARP1, PTEN, NFE2L2, SIRT1, NFKB1, PTGS2, BCL2, CASP3, ATF6, CASP9, EGFR, CASP1, JUN, HSP90AA1, MDR2, P2RX7, BSG, BIRC3, and BIRC2* (Figure 3B).

Based on the GO and KEGG analysis, the most enriched pathway in the biological process (BP) category was the response to oxygen‐containing compound, whereas in the cellular component (CC) category, it was the transcription regulator complex, and in molecular function (MF) category, it was identical protein binding (Figure 3C–E).

KEGG analysis was conducted in the DAVID database, and the top 10 most enriched pathways were analyzed and sorted according to the p‐value. The top 10 pathways are lipid and atherosclerosis, pathways in cancer, small cell lung cancer, apoptosis, AGE‐RAGE signaling pathway in diabetic complications, NF‐kappa B signaling pathway, toxoplasmosis, human cytomegalovirus infection, *TNF* signaling pathway, Salmonella infection (Figure 3F).

The intersection genes were analyzed in STRING for the PPI relationship, based on the combined score. The top 10 pairs of genes with high score and close interaction relationship are as listed as follows: *AKT1: HSP90AA1, CASP1: IL1B, EGFR: STAT3. EGFR: HSP90AA1, HSP90AA1: STAT3. HSP90AA1: TP53, JUN: NFE2L2, MDR2: TP53, PTEN: TP53, SIRT1: TP53* (Figure 3G). Subsequently, hub genes were screened based on degree centrality in Cytoscape, and the results identified *JUN, STAT3, CASP9, TP53, EGFR, AKT1, TNF, HSP90AA1, IL1B*, and *CASP3* as key targets within the network (Figure 3H).

#### 3.3.2 Construction of a relation for hub genes among PPI, GO, and KEGG

**Figure 4:**
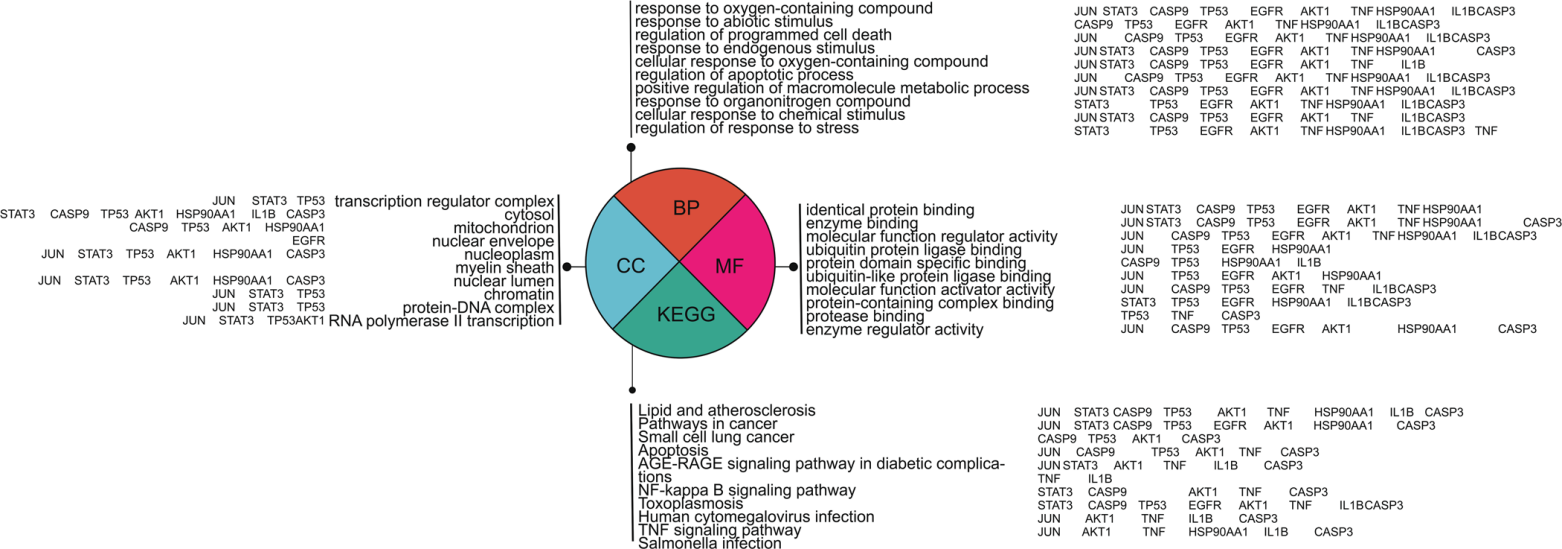
Hub gene‐enriched GO and KEGG pathways. This figure illustrates the enrichment of hub genes in BP, CC, MF, and KEGG pathways. Key findings include the enrichment of 10 hub genes in oxygen‐containing compound response (BP), 7 hub genes in the cytosol (CC), 9 hub genes in enzyme binding (MF), and 9 hub genes in lipid and atherosclerosis pathways (KEGG). BP, biological process; CC, cellular component; MF, molecular function.

In order to understand the relationships among hub gene, GO, and KEGG, hub genes were mapped onto enriched GO categories and KEGG pathways. We found that, in BP, all 10 hub genes were enriched in the response to oxygen‐containing compound pathway. In CC pathway, the cytosol enriched seven hub genes. For MF, 9 hub genes were mainly enriched in enzyme binding. In addition, in the KEGG pathway, nine hub genes were mainly enriched in lipid and atherosclerosis pathway (Figure 4).

#### 3.3.3 Molecular docking

**Figure 5:**
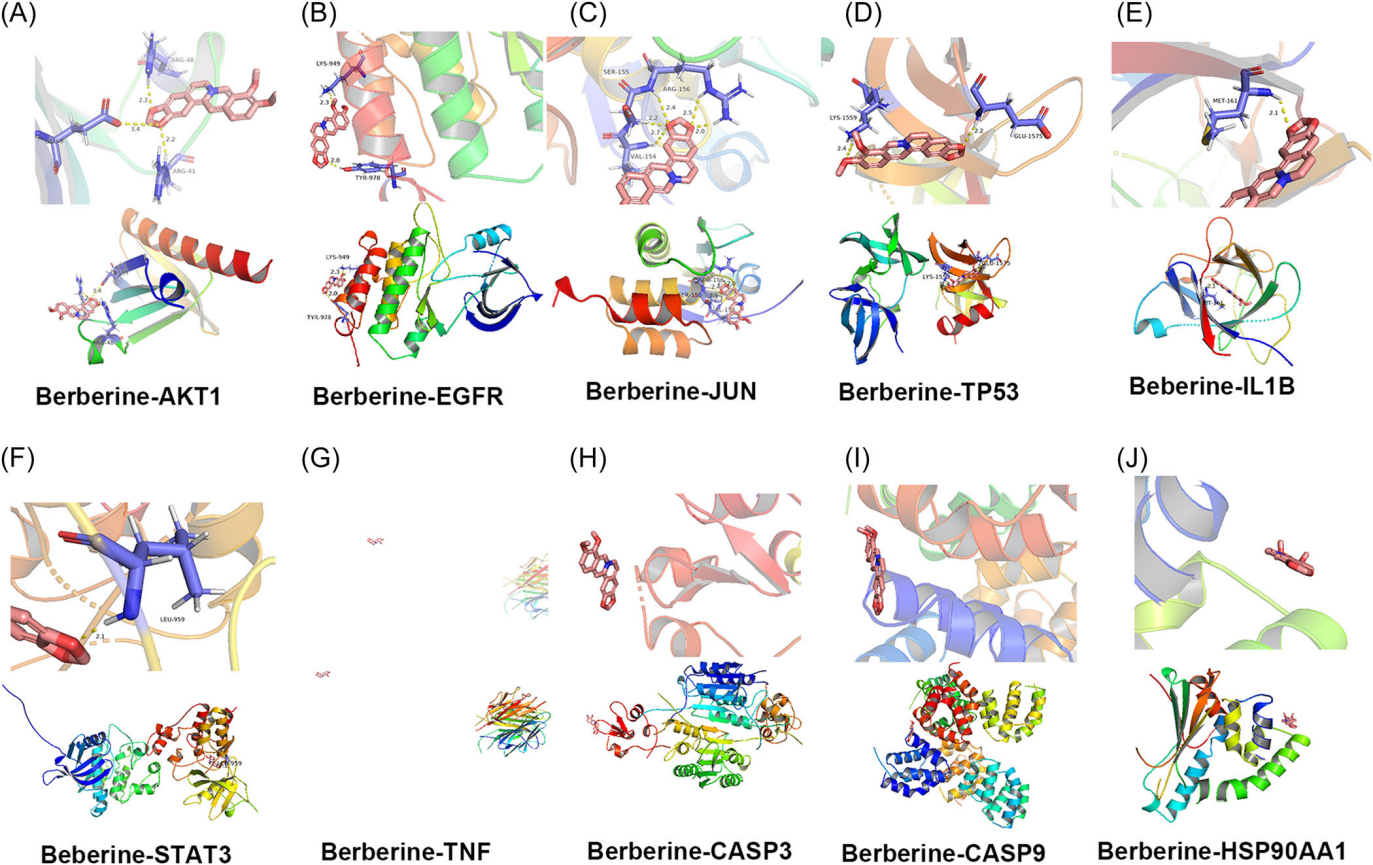
Molecular docking and site position. The molecular docking results and binding sites of berberine with different target proteins include AKT1 (A), EGFR (B), JUN (C), TP53 (D), IL1B (E), STAT3 (F), TNF (G), CASP3 (H), CASP9 (I), and HSP90AA1 (J).

To validate the direct relations between BBR and hub genes, molecular docking was performed. As a result, *AKT1, EGFR, JUN, TP53, IL1B*, and *STAT3* can successfully combine with BBR and form stable ligand–target complexes, while *TNF, CASP3, CASP9*, and *HSP90AA1* failed to form stable docking conformations with BBR, indicating the absence of direct binding interactions at their predicted active sites (Figure 5).

### 3.4 RT‐qPCR and Western blot validation

#### 3.4.1 Gene expression validation by RT‐ qPCR from animal models

**Figure 6:**
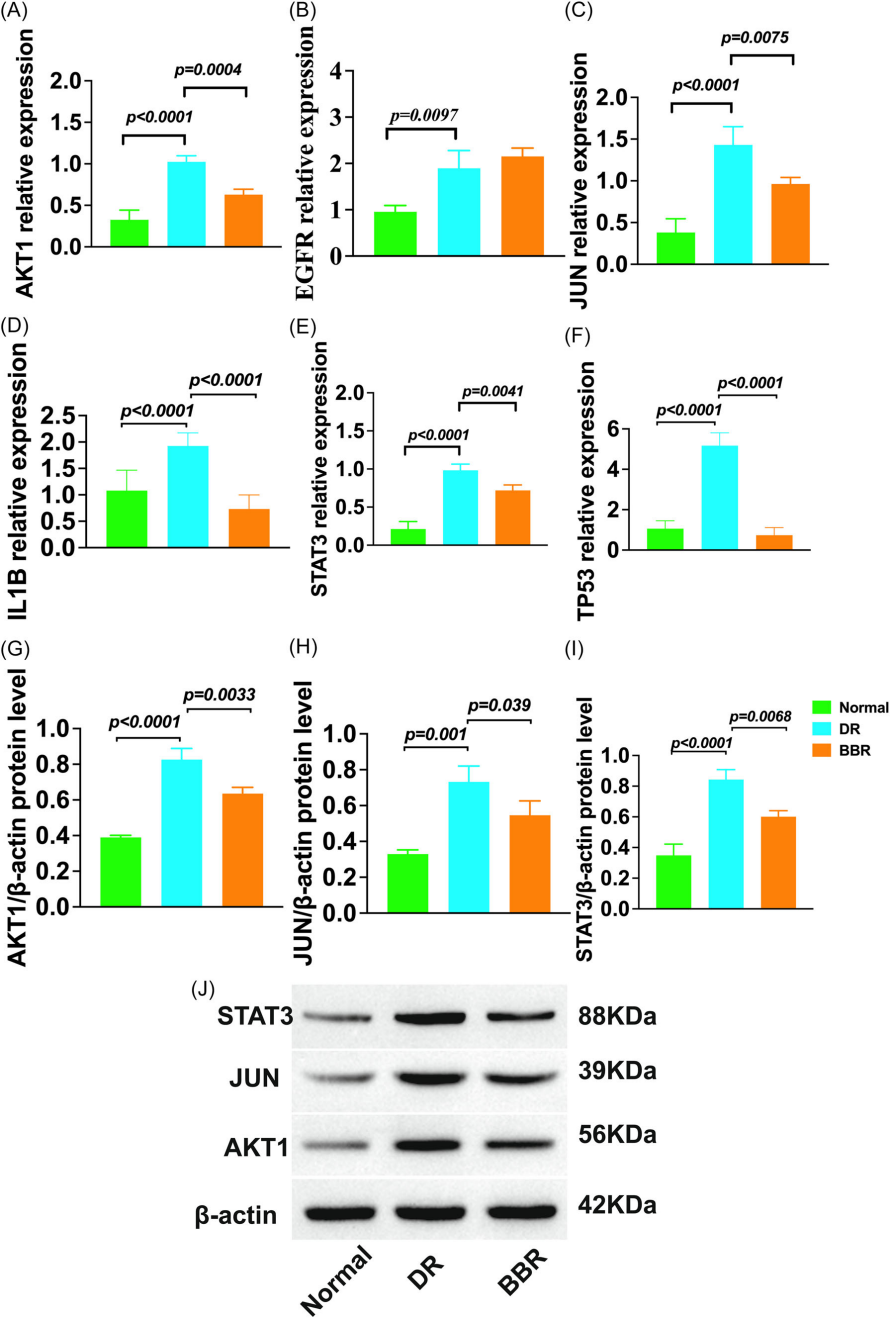
mRNA and protein expression levels of key genes across experimental groups. (A–F) RT‐qPCR analysis of relative mRNA expression of *AKT1* (A), *EGFR* (B), *JUN* (C), *IL1B* (D), *STAT3* (E), and *TP53* (F) in Normal, DR, and BBR groups. *n* = 3. (G–I) Western blot analysis of protein expression of AKT1 (G), JUN (H), and STAT3 (I) in Normal, DR, and BBR groups. *n* = 3. (J) Representative blots showing STAT1, JUN, AKT1, and β‐actin. Protein levels were normalized to β‐actin. BBR, berberine; DR, diabetic retinopathy.

To explore the potential molecular alterations in DR and BBR treatment, we analyzed the expression profiles of several key molecules. DR treatment significantly increased the expression levels of *AKT1* (*p* < 0.0001), *JUN* (*p* < 0.0001), *IL1B* (*p* < 0.0001), *STAT3* (*p* < 0.0001), and *TP53* (*p* < 0.0001) compared to normal controls. BBR treatment effectively reversed these DR‐induced changes, with *AKT1* (*p* = 0.0004), *JUN* (*p* = 0.0075), *IL1B* (*p* < 0.0001), *STAT3* (*p* = 0.0041) and *TP53* (*p* < 0.0001) showing significant reductions compared to DR. For *EGFR*, DR caused a significant increase compared to normal controls (*p* = 0.0097), but no significant difference was observed between DR and BBR treatments (Figure 6A–F).

#### 3.4.2 Verification of upstream signaling molecules by Western blot

As EGFR was not shown to have a significant positive effect by BBR, it was excluded from the subsequent study. Both upstream signaling molecules, IL‐1B and TP53, were also excluded as these molecules were not specifically related to DR and contributed more to tumor suppression, which is not the central focus of this research. Western blot analysis showed that the expression levels of STAT3, JUN, and AKT1 proteins differed significantly among the Normal, DR, and BBR‐treated groups. Compared to the Normal group, the DR group exhibited significantly elevated protein expression of AKT1 (*p* < 0.0001), JUN (*p* = 0.001), and STAT3 (*p* < 0.0001). However, BBR treatment significantly reduced the expression of AKT1 (*p* = 0.0033), STAT3 (*p* = 0.0068), and JUN (*p* = 0.039). These results suggest that the dysregulation of these proteins is linked to DR pathogenesis, and BBR may exert protective effects by modulating their expression (Figure 6G–J).

### 3.5 Effects of BBR and RNAi in high glucose‐induced RGC damage

#### 3.5.1 Cell viability

**Figure 7:**
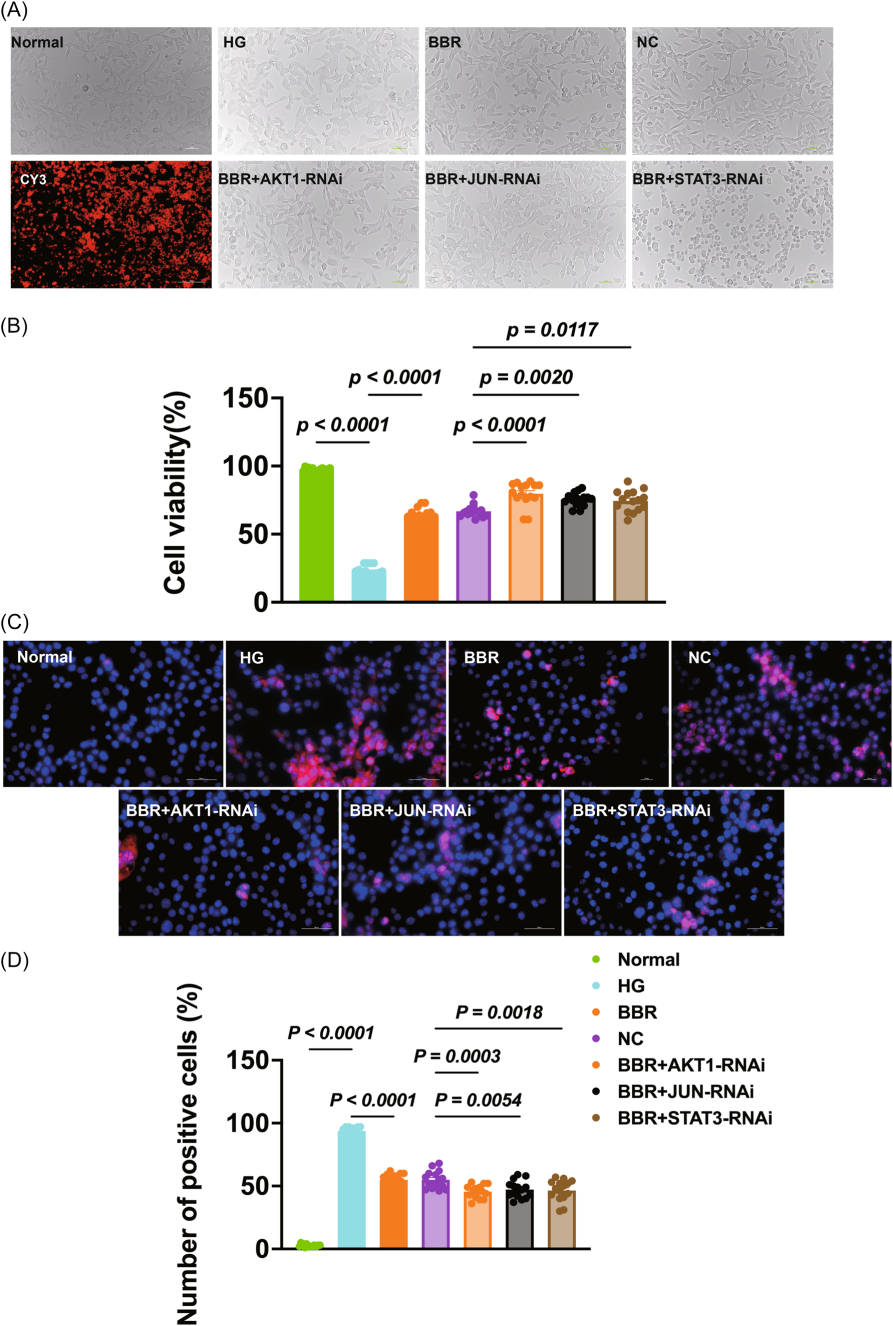
Cell viability and TUNEL staining in different groups. (A) Bright‐field images of cells (20×). (B) Cell viability detected by CCK‐8 assay. *n* = 5 wells per group with three replicates. (C) TUNEL staining images (red: TUNEL‐positive cells; blue: DAPI; 40×). (D) Quantification of TUNEL‐positive cells. *n* = 5 wells per group with three replicates. BBR, berberine; HG, high glucose; NC, negative control.

Cellular morphology was observed under bright‐field microscopy (Figure 7A). Normal group cells exhibited good adherence and plump morphology; the HG group showed significantly reduced cell numbers with partial cell shrinkage and detachment; the BBR and NC groups demonstrated improved cell status compared to the HG group; the BBR + AKT1‐RNAi, BBR + JUN‐RNAi, and BBR + STAT3‐RNAi groups showed increased cell numbers compared to the NC group. CCK‐8 assay results revealed that cell viability in the HG group was significantly decreased compared to the Normal group (*p* < 0.0001), which was significantly restored after BBR treatment (*p* < 0.0001). Compared with the NC group, cell viability was elevated in the BBR + AKT1‐RNAi group (*p* < 0.0001), the BBR + JUN‐RNAi group (*p* = 0.0020), and the BBR + STAT3‐RNAi group (*p* = 0.0117) (Figure 7B).

#### 3.5.2 TUNEL staining

TUNEL staining revealed an increased number of positive cells in the HG group relative to the Normal group (Figure 7C). Both the BBR and NC groups exhibited markedly reduced red fluorescence signals compared to the HG group. BBR + AKT1‐RNAi, BBR + JUN‐RNAi, and BBR + STAT3‐RNAi groups demonstrated further reductions in red fluorescence signals versus the NC group. Quantitative analysis (Figure 7D) indicated that TUNEL‐positive cells were significantly elevated in the HG group compared to the Normal group (*p* < 0.0001), which was substantially decreased following BBR treatment (*p* < 0.0001). Compared with the NC group, positive cells were further reduced in BBR + AKT1‐RNAi (*p* = 0.0003), BBR + JUN‐RNAi (*p* = 0.0054), and BBR + STAT3‐RNAi (*p* = 0.0018) groups. These findings indicate that silencing *AKT1*, *JUN*, and *STAT3* genes potentiates the protective effects of BBR.

## 4 DISCUSSION

DR, as a type of complication of diabetes that occurs on the retina, generally induces severe diminution of vision. The progression of DR involved various pathophysiological pathways, including oxidative stress, inflammation, stimulation of vascular growth factor in the eye, subtypes of protein kinase C, and activation of the hexosamine pathway. Complications of eye diseases, resulting from the structural design and physiological disorders of the eyes, often impede the treatment effectiveness.^20^ Current treatments include the usage of ITREAL anti‐vascular endothelial growth factor (VEGF), glucocorticoid implantation, laser, and surgery, which have drawbacks such as vision loss and risk of infections. Therefore, alternative treatment options are currently being pursued to address more effective outcomes of DR management and treatment.

In this study, a DR model was established by intraperitoneally injecting STZ into rats, while control rats received intraperitoneal injections of vehicle solution. All rats were maintained for 2 months, during which time the STZ‐treated rats developed retinal damage, including pathological changes in the retinal pigment epithelial cells, outer nuclear layer, inner nuclear layer, and retinal ganglion cells. The retinal changes were assessed using H&E staining, Nissl staining, and immunofluorescent staining, and the underlying molecular mechanism was investigated using network pharmacology and experimental validation, in which *AKT1, JUN*, and *STAT3* could be considered as vital targets. Our findings provided crucial evidence for the usage of BBR in the treatment of DR and explained the core mechanism between BBR and its direct targets.

Previous reports have shown that immunofluorescence staining revealed significantly increased expression of IL‐1β, IL‐18, GSDMD, Caspase‐1, and NLRP3 in the DR model group compared to the normal group.^21^ In addition, BBR has been reported to inhibit oxidized low‐density lipoprotein (ox‐LDL)‐induced pyroptosis and inflammatory response of macrophages, possibly through the regulation of NLRP3/caspase‐1/GSDMD pathway and the downregulation of the expression of inflammatory cytokines IL‐18 and IL‐1β.^22^ In this study, using immunofluorescence staining, we mapped the localization of pyroptosis‐related proteins in the retina and determined their changes following BBR administration, which demonstrated that cell pyroptosis occurred in RGCs after DR induction and that berberine treatment effectively protected RGCs by modulating the expression of several genes associated with pyroptosis markers.

Network pharmacology identified 28 overlapping genes among BBR, DR, and pyroptosis‐related genes. Ten key hub genes (*JUN, STAT3, CASP9, TP53, EGFR, AKT1, TNF, HSP90AA1, IL1B, CASP3*) were identified in the PPI network. GO analysis suggested BBR may treat DR by regulating oxidative stress, transcriptional activity, and protein binding. KEGG analysis showed pathways like lipid metabolism and atherosclerosis, linking DR and atherosclerosis via shared mechanisms such as oxidative stress and the AGE‐RAGE pathway,^23^, ^24^ with retinal microvascular damage as an early sign of macrovascular disease. Molecular docking revealed six hub genes (*AKT1, EGFR, JUN, TP53, IL1B, STAT3*) as potential direct targets of BBR.

*AKT1* is a serine protein kinase. Studies suggest that it helps protect against DR by reducing inflammation and alleviating retinal abnormalities caused by diabetes.^25^ *EGFR* is involved in the process of neovascularization in DR by regulating *VEGF* expression, and studies have shown that *EGFR* inhibition reduces retinal neovascularization.^26^, ^27^ *JUN*, a putative transforming gene of avian sarcoma virus 17, encodes a protein that is highly similar to the viral protein and induces cell death. TP53 is upregulated in DR and may play a key role in retinal ganglion cell apoptosis. As a crucial tumor suppressor, TP53 regulates cell cycle, DNA repair, and apoptosis under cellular stress.^28^ In contrast, STAT3 is a protein‐coding gene that plays a central role in cell growth and survival signaling. Alterations in STAT3 expression or activation have also been implicated in the regulation of pyroptosis. Together, by molecular docking combined with RT‐qPCR test, we confirmed that *AKT1, JUN, IL‐1β, TP53*, and *STAT3* could be designated as the crucial and core genes in the process of BBR administration for DR protection induced by DR. *IL‐1β* expression has been confirmed in our previous study^15^ and *TP53's* involvement in DR is less established compared to its primary role in tumor suppression. *JUN, STAT3*, and *AKT1* were prioritized for further Western blot validation as they represent key nodes in signaling pathways with well‐documented roles in DR pathogenesis.

Additionally, Western blot analysis was performed to validate the protein‐level changes of three key signaling molecules, including AKT1, JUN, and STAT3. As critical regulators of cell survival, inflammatory response, and cell death pathways, these signaling molecules exhibited significant expression changes in DR models. Western blot results showed that BBR treatment significantly attenuated the DR‐induced overexpression of AKT1, JUN, and STAT3, further validating them as important therapeutic targets of BBR.

The significant changes in expression levels of these genes, as verified through experimental detection, coupled with the stable binding conformations revealed through molecular docking, strongly suggest that BBR exerts its therapeutic effects through modulating multiple signaling pathways. These genes collectively form a regulatory network involved in cell survival, inflammatory response, and pyroptosis, providing a molecular basis for understanding BBR's protective effects in DR treatment.

Furthermore, we have found that the cell viability of RGCs significantly improves following BBR treatment. This suggests that BBR can improve cellular function under diabetic conditions by reducing oxidative stress and inflammatory responses.^29^ Furthermore, the combined treatment of RNAi targeting the *AKT1, JUN*, and *STAT3* genes further enhances BBR's protective effects, indicating that these genes play critical roles in the pathway of high‐glucose‐induced cellular damage.

The synergistic effect of BBR and RNAi in improving cell viability suggests that targeting and inhibiting these signaling molecules may amplify the beneficial effects of BBR. These findings provide important insights into the molecular mechanisms of high‐glucose‐induced retinal damage and propose a novel therapeutic strategy combining BBR with gene‐targeting approaches for the treatment of DR.

However, although BBR shows therapeutic potential in laboratory studies, its clinical application is limited by extremely low oral bioavailability (<1%) due to poor intestinal absorption and first‐pass metabolism. Its complex pharmacokinetics involve cytochrome P450 enzymes (CYP2D6, CYP1A2) and uridine diphosphate‐glucuronosyltransferase enzymes (UGT2B1, UGT1A1). Short‐term safety is acceptable, but long‐term data are lacking, with some patients experiencing initial gastrointestinal discomfort. Variability in formulations and the lack of standardized dosing further restrict its use. Future efforts should aim to enhance its solubility, absorption, and formulation.^30^

## 5 CONCLUSION

BBR alleviates retinal inflammation in diabetic conditions by targeting pyroptosis and regulating key signaling molecules AKT1, JUN, and STAT3. These findings highlight its potential in treating DR and provide insights into its pharmacological mechanisms.

## AUTHOR CONTRIBUTIONS

Na Li wrote the main manuscript text, analyzed the data, and conducted/organized the experiments. Ji‐Lin Chen, Yi‐Jian Sun, and Jia‐Fan Sun contributed to experimental validation and data acquisition and assisted in data interpretation. Fatin Athirah Pauzi, Song‐Lin Zhu, Amy Yi Hsan Saik, and Alan Han‐Kiat Ong critically revised the manuscript for important intellectual content. All authors reviewed and approved the final manuscript.

## CONFLICT OF INTEREST STATEMENT

The authors declare no conflicts of interest.

## ETHICS STATEMENT

This study was conducted in accordance with the Animal Research: Reporting of In Vivo Experiments (ARRIVE) guidelines. All procedures were approved by the Ethics Committee of Kunming Medical University (Approval No. KMMU20242021). Additionally, the use of animals in this research was approved by the UTAR Scientific and Ethical Review Committee (SERC) (Approval No. U/SERC/58‐29/2024).

## Supporting information

Supplementary_material.

## Data Availability

All data are included in this manuscript.
