# Supplementary material for: Berberine alleviates pyroptosis of retinal ganglion cells in diabetic retinopathy by regulating AKT1, JUN, and STAT3
Source: Ibrain. 2026 Mar 26;12(2):222–37. doi: 10.1002/ibra.70018 (PMC13310242; doi:10.1002/ibra.70018)
Supplement: Supplementary file 1 — Supplementary_material. [file IBRA-12-222-s001.docx]

Supplementary material

**Supplementary Table 1. Partial genetic list of diabetic retinopathies**

| INS | AKT2 | IGF1 | EDN1 | NOS2 | TTC8 | IFIH1 |
| --- | --- | --- | --- | --- | --- | --- |
| GCK | BLK | LEP | CTNNB1 | CP | BBS4 | MMP2 |
| KCNJ11 | PTPN22 | CRP | SLC2A1 | UCP3 | RLBP1 | IMPG2 |
| HNF1A | IRS2 | CCL2 | IAPP | AGT | CISD2 | CXCL8 |
| ABCC8 | GLIS3 | ICAM1 | SUMO4 | MIR17 | MMP9 | IGF2 |
| HNF4A | EPO | HLA-DRB1 | IL1B | MIR155 | KDR | BBS7 |
| INSR | MTNR1B | MIR29C | CDKAL1 | RPE65 | HIF1A | MIR192 |
| HNF1B | LMNA | HLA-DQA1 | SAG | SERPINF1 | DPP4 | FZD4 |
| PDX1 | MT-TL1 | MTHFR | LRP5 | PIK3R1 | CEP290 | MIR126 |
| WFS1 | CEL | RHO | FLT1 | TULP1 | TP53 | MT-CO1 |
| PPARG | ADIPOQ | MC4R | GAD2 | PRKCB | GLP1R | ADRB2 |
| IL6 | ALMS1 | APOE | APOB | APOA1 | SOD1 | CXCL12 |
| NEUROD1 | ZFP57 | PTF1A | MT-ATP6 | FGF2 | SELE | PPARGC1A |
| VEGFA | TBC1D4 | IL2RA | GATA6 | SORD | RBP4 | MKS1 |
| PAX4 | AKR1B1 | PLAGL1 | MT-ND1 | MIR145 | SCAPER | IL18 |
| TCF7L2 | TNF | RFX6 | REN | ANGPT2 | BBIP1 | HHEX |
| ACE | PTPN1 | GCG | AGTR1 | TLR4 | AOC3 | RPGR |
| IRS1 | ALB | MIR21 | TGFB1 | LPL | MEN1 | CCN2 |
| AVPR2 | SOD2 | ABCA4 | DNAJC3 | USH2A | HAMP | PPARA |
| SLC30A8 | FOXP3 | HFE-AS1 | PTPRN | AKT1 | GHRL | PROM1 |
| RETN | CTLA4 | IL1RN | CDKN2A | UCP2 | IGFBP1 | CFAP418 |
| SLC2A2 | HLA-DQB1 | HYMAI | ADRB3 | C12orf43 | BDNF | IGF1R |
| AVP | HMGA1 | MT-TK | CAV1 | VCAM1 | HP | POLG |
| KLF11 | MT-TE | EIF2AK3 | BEST1 | VDR | CRB1 | RDH12 |
| ENPP1 | CAPN10 | STAT3 | MLKL | PRPH2 | NEUROG3 | PGF |
| INS-IGF2 | MIR29A | CAT | VWF | IGFBP3 | NR2E3 | NDP |
| IGF2BP2 | APPL1 | LEPR | CCR5 | ARL6 | BBS10 | HBA1 |
| PON1 | DCAF17 | BBS2 | IL10 | AIRE | AR | CFTR |
| HFE | AGER | ITPR3 | SST | FTO | GCGR | R3HDRL-AS1 |
| TNFRSF11B | NOS3 | SERPINE1 | BBS1 | GAD1 | CDKN2B | NAGLU |

**Supplementary Table 2. List of Berberine genes**

| LDLR | MMP2 | HNF1A | CYBB | ATF3 | LPL | DUX4 |
| --- | --- | --- | --- | --- | --- | --- |
| BCL2L1 | PRKAA1 | MAPK8 | PPIF | GCKR | F2 | MTOR |
| CCND1 | JUN | GDF15 | CD69 | ENDOG | FASN | INSR |
| CDK4 | NFKB1 | CYP3A4 | CASC2 | HNF4A | HDAC1 | CYP2A6 |
| SLC2A4 | BID | STAT3 | CAMKK2 | CASP1 | LEP | PKD1 |
| CASP8 | NFKBIA | BAX | NDUFS4 | ABCC1 | SPP1 | NME1 |
| CDK6 | PPARG | HIF1A | MAPT | IRS1 | LIPE | LSR |
| CDK2 | ANXA5 | STK11 | KCNJ11 | AKR1B1 | SIRT3 | PRKCD |
| WEE1 | PRKAA2 | CDKN1A | RAD52 | TIMP2 | RUNX2 | TLR4 |
| CDK1 | MAPK1 | EZR | SENP8 | AEBP1 | F3 | SRC |
| CYP2D6 | PARP1 | PTEN | DPP4 | PDCD4 | ATIC | RELA |
| CCNB1 | MMP9 | BCHE | PIK3C3 | CSN1S1 | SOCS1 | MAPK10 |
| BIRC5 | IKBKB | MIR93 | HSPA5 | PTGS2 | BCL2L2 | PLCB1 |
| BCL2 | PLAU | EGFR | ATF6 | SLC22A2 | KCNH6 | PLCB3 |
| CYCS | PRKAB1 | MMP1 | MSR1 | SLC22A1 | DAXX | HSPG2 |
| CYP1A2 | NOS2 | CBL | GADD45A | MT-CO2 | HIF3A | PLCG1 |
| TNF | MIR21 | CDC42 | PLIN2 | LOC110806262 | BGLAP | MAP3K5 |
| MAPK14 | GATA3 | RAC1 | XAF1 | PIK3CA | CLIP2 | PLCB4 |
| TERT | GATA2 | MCL1 | TAS2R38 | KCNH2 | ANGPTL2 | P2RX7 |
| SLC6A4 | IL6 | ID1 | CHUK | PREP | PTPA | SREBF1 |
| TP53 | RAD51 | BCAP31 | GCK | FOXO3 | MIR122 | NDUFS6 |
| AKT1 | DDIT3 | KDR | DHFR | OLR1 | MIR17 | HDC |
| SARM1 | SIRT1 | ABCG2 | INS | HOTAIR | MIR203A | BSG |
| NFE2L2 | ABCC8 | IL2RA | TYMS | MIR34A | MIR429 | KHSRP |
| CCL2 | HNRNPD | RXRA | BIRC3 | MIR99A | TOP1 | EXOC7 |
| IL1B | TGFB1 | CDKN2B | VCAM1 | MIR125B1 | ACHE | CCL19 |
| CASP3 | CYP1A1 | ABCA1 | HYOU1 | NOS3 | DICER1 | FBN1 |
| PCSK9 | AHR | VEGFA | SAT1 | ADA | MYC | HSF1 |
| ABCB1 | AR | NR1H3 | CFLAR | TNFRSF10B | MDH2 | AKR1A1 |
| CXCL8 | SLC2A1 | CASP9 | BIRC2 | EP300 | EGR1 | DRD1 |
| NR3C1 | GLUD1 | NPY | NTS | PTPRC | XDH | ATP2A2 |
| CHEK2 | CAT | ITGAL | FFAR1 | PTPN1 | KITLG | FAS |
| CTNNB1 | CACNA1C | ADRA2B | FKBP1B | CD4 | HTR4 | FN1 |
| MDR2 | KCNQ1 | DRD3 | LOC110596866 | ROR2 | SMURF1 | XIAP |
| FOS | GSK3B | COX5A | CYB5A | HTR7 | CRHR1 | CD9 |
| GRIN2B | CHRM3 | HSF2 | MIR212 | HTR3A | CXCL12 | ANKK1 |
| SCN5A | NQO1 | SETD7 | KIT | SMAD6 | HTR6 | IGHE |
| SOD1 | HSP90AA1 | DNASE1 | ACVR1 | BACE1 | SMAD1 |  |
| TH | GLI2 | IFNA2 | APP | CD44 | CD2 |  |

**Supplementary Table 3. List of pyroptosis genes**

| GSDRD | TXNIP | PGF | ADORA2B | TNF | POP1 | CXCL8 |
| --- | --- | --- | --- | --- | --- | --- |
| GSDRE | DDX3X | NLRX1 | ADORA2A | VIM | LINC00958 | IL13RA2 |
| NLRP3 | MIR22 | SLC16A4 | ADORA3 | CAPN1 | MIR4306 | STXBP3 |
| CASP1 | MIR125A | IL32 | METTL3 | JUN | MDR2 | GPER1 |
| GSDRC | GBP1 | CHRFAM7A | PECAM1 | RIPK3 | BTK | BST2 |
| GSDRB | MEG3 | MIR21 | TRIM31 | MIR139 | BCL2 | LYST |
| CASP4 | MIR135B | MIR124-1 | METTL14 | BHLHE40 | IL1RN | VPS28 |
| NLRP1 | MIR556 | MIR195 | MIR25 | BHLHE41 | RIPK1 | NCR1 |
| GSDRA | GJA1 | MIR485 | IFI16 | ALK | YWHAE | IL27 |
| GZMB | UBR2 | MALT1 | CAMP | TFAP2A | HSP90AA1 | SEC22B |
| CARD8 | CPTP | TLR2 | MRE11 | BIRC3 | ANXA2 | SIGLEC14 |
| CASP8 | PRDR1 | GSK3B | FNDC4 | E2F4 | NEDD4 | CLEC5A |
| GZMA | MIR214 | STK4 | FNDC5 | BIRC2 | HSP90AB1 | CGAS |
| IL1B | GAS5 | PTGS2 | ELANE | UBE2D2 | IRAK3 | MIR20B |
| DPP9 | TP53 | MST1 | PARP1 | LY96 | MELK | MIR15A |
| DPP8 | VDR | PRF1 | TRIM21 | GLMN | YWHAZ | FOXO3 |
| NLRC4 | PCSK9 | TRIM24 | PRKN | IRGM | STXBP2 | MIR30C1 |
| AIM2 | BRD4 | ELAVL1 | GBP5 | SCAF11 | UBE2D3 | NFE2L2 |
| CASP5 | IKBKE | MPEG1 | NR1H2 | NLRP13 | TLR9 | NEK7 |
| ZBP1 | AGER | MIR204 | CTSG | ADAMTS9-AS2 | CD14 | MIR23A |
| PYCARD | PKM | HOTTIP | MKI67 | NINJ1 | IFIH1 | KLF3-AS1 |
| CASP3 | CRTAC1 | CDKN2B-AS1 | IL36G | TUBB6 | GSTO1 | CEBPB |
| NAIP | TET2 | MIR9-1 | IL36B | MYD88 | HUWE1 | BSG |
| CASP6 | CTSV | MIR9-2 | NLRP6 | TLR8 | TNFSF13B | TFAM |
| DHX9 | UTS2 | MIR9-3 | PRTN3 | APAF1 | BECN1 | STING1 |
| NLRP9 | MIR155 | MIR497 | SERPINB1 | NOS1 | CHI3L1 | HNP1 |
| APIP | MLKL | EPHA2 | DUOX1 | NOS2 | RAB5A | PTEN |
| IL18 | NFKB1 | ABL1 | APOL1 | PKN2 | PANX1 | DRD2 |
| HMGB1 | APOE | HDAC6 | MEFV | DPEP1 | IL13 | ADORA1 |
| STAT3 | SDHB | SQSTM1 | FOXP3 | CHMP1A | ASIC1 | MIR103A2 |
| MALAT1 | P2RX7 | IRF3 | NLRP7 | PYDC2 | BRCC3 | MIR103A1 |
| SIRT1 | EEF2K | CDK9 | ANO6 | ACE2 | ATG7 | FADD |
| KCNQ1OT1 | CD274 | TREM1 | BNIP3 | AKT1 | LRPPRC | VCAM1 |
| TREM2 | FGF21 | TSLP | XIST | EGFR | ERP44 | SESN2 |
| MIR223 | DLX6-AS1 | ZDHHC1 | MIR107 | TP63 | CDC37 | ATF6 |
| IRF1 | TRPM2 | CHMP4B | PDCD6IP | VPS4B | ATG3 | CASP9 |
| IRF2 | ORMDL3 |  |  |  |  |  |
